# Explicit Compression Degradation Estimations for Low‐Sampling Single‐Pixel Imaging using Hadamard Basis

**DOI:** 10.1002/advs.202512655

**Published:** 2025-11-30

**Authors:** Haoyu Zhang, Jie Cao, Chang Zhou, Haifeng Yao, Qun Hao

**Affiliations:** ^1^ School of Optics and Photonics Beijing Institute of Technology Beijing 100081 China; ^2^ Yangtze Delta Region Academy Beijing Institute of Technology Jiaxing 314003 China; ^3^ National Key Laboratory on Near‐Surface Detection Beijing 100072 China; ^4^ Changchun University of Science and Technology Changchun 130022 China

**Keywords:** compressive sensing, computational imaging, degradation estimation, self‐supervised learning, single‐pixel imaging

## Abstract

Single‐pixel imaging (SPI) is a promising imaging modality that enables 2D image acquisition using 1D photocurrent measurements. In SPI, the number of measurements strongly restricts image quality. Compressive sensing methods allow SPI reconstruction using undersampled measurements. Recent studies have focused on restoration schemes using implicit prior assumptions or data‐driven approaches. However, explicit compression degradation models for SPI are still unclear. Here, a degradation estimation technique is presented to explicitly describe compressive sampling for low‐sampling SPI reconstruction using Hadamard basis patterns. The compression degradation models are reflected by the results at different sampling ratios. A self‐supervised learning method is proposed to estimate explicit degradation models, which are mainly composed of blur kernels. Blur kernels varying with sampling ratios and corresponding SPI results are numerically and experimentally demonstrated. Furthermore, this approach is demonstrated for single‐pixel video imaging in dynamic scenes. It is anticipated that the compression degradation estimation technique will further promote the practical application of SPI.

## Graphical abstract

This study proposes a degradation estimation technique to explicitly describe compressive sampling for low‐sampling Hadamard single‐pixel imaging. Blur kernels in explicit degradation models are estimated by the self‐supervised learning method without labeled data and implicit priors. Results of single‐pixel simulations and experiments are restored by using the deconvolution network and the kernel estimations.

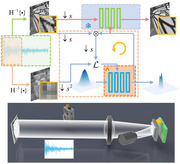

## 1 Introduction

In contrast to conventional cameras featuring abundant pixels, single‐pixel imaging (SPI) achieves 2D imaging through the correlation between 2D modulation patterns and 1D bucket signal. It utilizes a digital micromirror device (DMD) to perform light modulation and a bucket detector to receive total intensity. Relying on bucket detectors, SPI has advantages over conventional imaging modalities in terms of wavelength, sensitivity and cost^[^ ^1^ ^]^. Single‐pixel camera architectures have been widely applied in various fields such as microscopy,^[^ ^2^, ^3^ ^]^ X‐ray imaging,^[^ ^4^, ^5^ ^]^ holography,^[^ ^6^, ^7^ ^]^ mid‐infrared imaging,^[^ ^8^ ^]^ UV imaging,^[^ ^9^ ^]^ high‐speed imaging^[^ ^10^, ^11^ ^]^ and 3D imaging.^[^ ^12^, ^13^, ^14^ ^]^

When imaging with a resolution of *N* × *N*, the number of fully sampled measurements *M* is equal to the number of total pixels *N* ^2^ in principle. Insufficient measurements result in degraded images at a sampling ratio δ. The reconstruction process as an inverse problem is ill‐posed, which can be resolved by compressive sensing.^[^ ^15^ ^]^ In general, recent SPI methods can be classified into two categories:^[^ ^16^ ^]^ data‐driven methods requiring training samples, and model‐driven methods using implicit prior information. The former category often employs massive simulation samples to train end‐to‐end neural networks, which non‐iteratively recover images through constant model parameters. Fueled by the learning ability of neural networks with various model architectures,^[^ ^17^, ^18^, ^19^, ^20^, ^21^, ^22^, ^23^ ^]^ data‐driven methods are treated as black boxes, with limited interpretability in physics. This category achieves impressive reconstruction performance, but the domain gap problem between labeled data and test data exists in the methods. Furthermore, deep reinforcement learning^[^ ^24^ ^]^ is introduced into SPI for using a few training samples. On the other hand, model‐driven methods iteratively perform image restoration without any labeled data. Several works incorporate implicit priors (e.g., total variation,^[^ ^15^ ^]^ sparsity,^[^ ^25^ ^]^ deep image prior,^[^ ^26^, ^27^ ^]^ and deep generative prior^[^ ^28^ ^]^) into the SPI reconstruction framework. However, most model‐driven methods are supported by implicit prior assumptions instead of explicit compression degradation models.

In this work, we present explicit compression degradation estimations for the SPI reconstruction technique based on the Hadamard basis. Compared to random coding approaches that bring high‐frequency noise caused by undersampling, Hadamard SPI acquiring spectrum coefficients enables compressive imaging with less noise. Nonetheless, the compression process still causes the loss of pixel information and mosaic artifacts. The compression degradation models are described by convolution operations with blur kernels, which can be reflected in results at different sampling ratios. We estimate blur kernels using a self‐supervised neural network without labeled data and implicit priors. We show that compression degradation estimations are employed to infer SPI results by adopting an off‐the‐shelf deconvolution network without training via Hadamard SPI datasets. Our results reveal that our approach can extract blur kernels of explicit compression degradation models and employ blur kernels for image restoration. Moreover, we demonstrate that our approach is used in single‐pixel video imaging for dynamic scenes, offering great potential for SPI reconstruction.

## 2 Results

### 2.1 Explicit Compression Degradation Models

**Figure 1:**
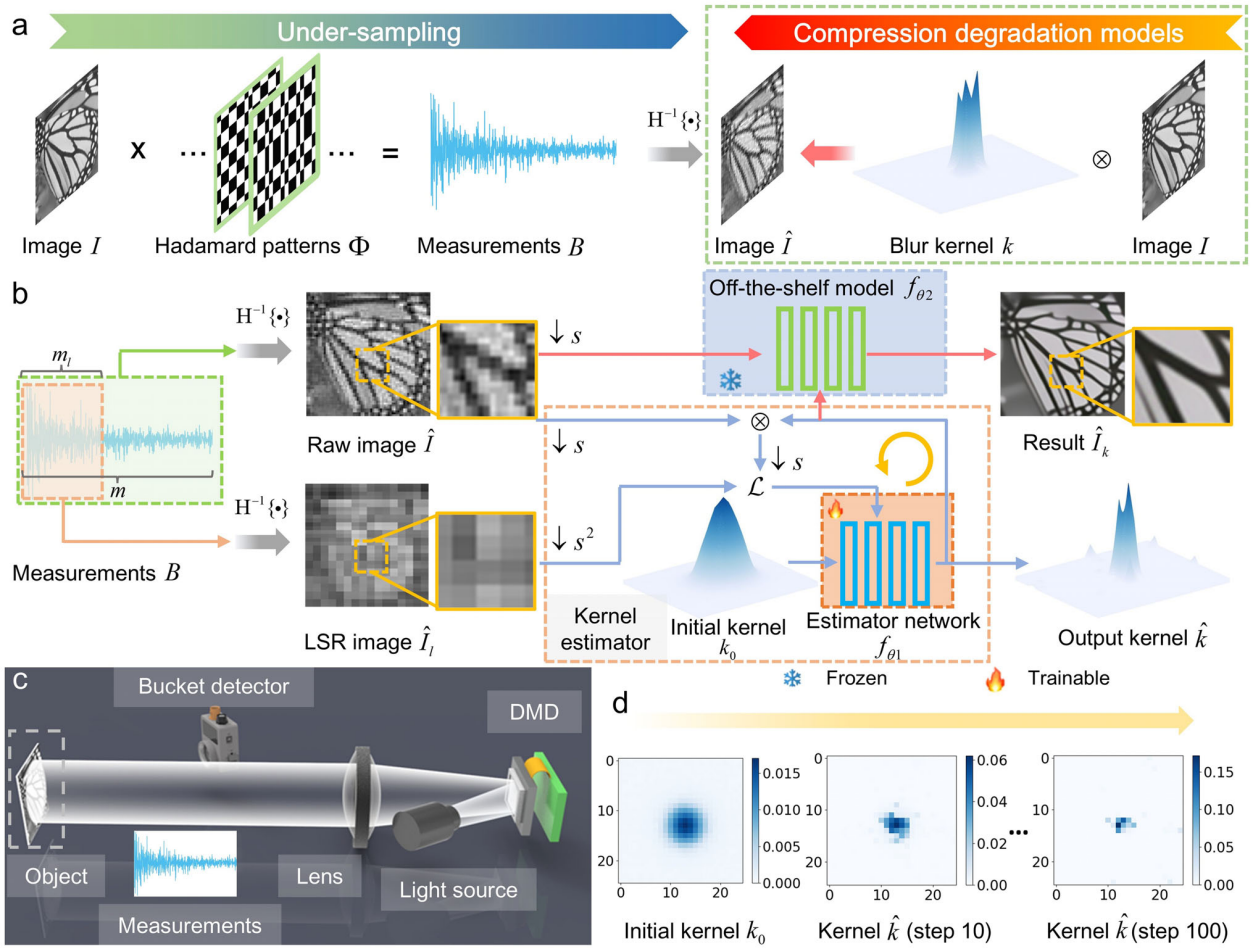
Concept of explicit compression degradation models for SPI using Hadamard basis. a) Schematic diagram of the proposed compression degradation models for SPI with undersampling. b) Workflow of estimating blur kernels based on a self‐supervised learning method. Output kernels are used to restore estimated images based on the off‐the‐shelf deconvolution network. c) Physics model of SPI using structured illumination schemes for imaging validation. d) Predicted blur kernels at iteration steps from 1 to 100.

As illustrated in **Figure** **1**a, an image *I* is modulated by Hadamard patterns Φ with orthogonality for single‐pixel acquisition. The intensity measurements *B* are generated by the inner product between Hadamard patterns and object images. Spatial information from low to high frequencies is sequentially captured. A raw image $\hat{I}$ can be restored by inverse Hadamard transform H^−1^{ · } using *m* under‐sampled measurements *B* and Hadamard basis patterns. Owing to insufficient 1D measurements in the sampling process, the directly restored images are degraded. Only low‐frequency information is measured by a few Hadamard patterns, which results in a blurring problem. Inspired by the concept of degradation models in super‐resolution issues^[^ ^29^, ^30^, ^31^, ^32^ ^]^ with mosaic reconstructions, compressive sampling of Hadamard SPI is described by explicit compression degradation models with blur kernels *k* varying with sampling ratios. The degraded images from insufficient measurements can be regarded as the ground truth after convolution with blur kernels *k*. Mathematically, compression degradation models for Hadamard SPI are expressed as follows:

(1) $$\hat{I}=U\left(I_{↓s}⊗k\right)+\gamma$$

where ⊗ represents the convolution operation, ↓*s* denotes the s‐fold resolution reduction caused by compressive sampling, U(·) represents the pixel expansion operation for ensuring consistency with the pixel number of fully sampled results, and γ denotes noise. The reduction factor *s* in our work is constrained by *s* = 2 or 4, which can be chosen to mitigate mosaic artifacts influencing blur kernel estimations (see Note S1, Supporting Information).

Without additional training data and implicit priors, we present a self‐supervised learning method to estimate blur kernels in compression degradation models. Normally, the degradation models are calculated by a compressed raw image $\hat{I}$ at a sampling ratio of δ and the ground truth. However, it is difficult to obtain the ground truth from insufficient measurements. As depicted in Figure 1b, we use the raw image $\hat{I}$ from *m* measurements and the lower‐sampling‐ratio (LSR) image ${\hat{I}}_{l}$ from *m_l_* measurements to train neural networks relying on self‐supervision. With *m_l_* = *m* · δ, the raw image $\hat{I}$ as a slightly degraded reference and the LSR image ${\hat{I}}_{l}$ as a doubly degraded reference are directly recovered by inverse Hadamard transform, which can reflect approximate degradation processes. When performing SPI with a sampling ratio of δ, the sampling ratios of raw images and LSR images are respectively δ and δ^2^. The reconstruction scheme without using fully sampled results consists of two parts, including a kernel estimator with trainable parameters and an off‐the‐shelf deconvolution network with frozen parameters. In the kernel estimator, the initial state of kernels is optimized by a Markov Chain Monte Carlo sampling strategy.^[^ ^33^ ^]^ The parameters of the estimator network *f*_θ1_ are iteratively optimized by minimizing the loss function between LSR images and raw images. Image downsampling operations in the estimator are performed by keeping the upper‐left pixel in each image patch. Finally, we employ the off‐the‐shelf deconvolution network *f*_θ2_ based on deep unfolding architectures^[^ ^34^ ^]^ to generate results of the physics model shown in Figure 1c, which utilizes the kernel $\hat{k}$ from the iteration process illustrated in Figure 1d. More details are provided in the Experimental Section.

### 2.2 Simulations

**Figure 2:**
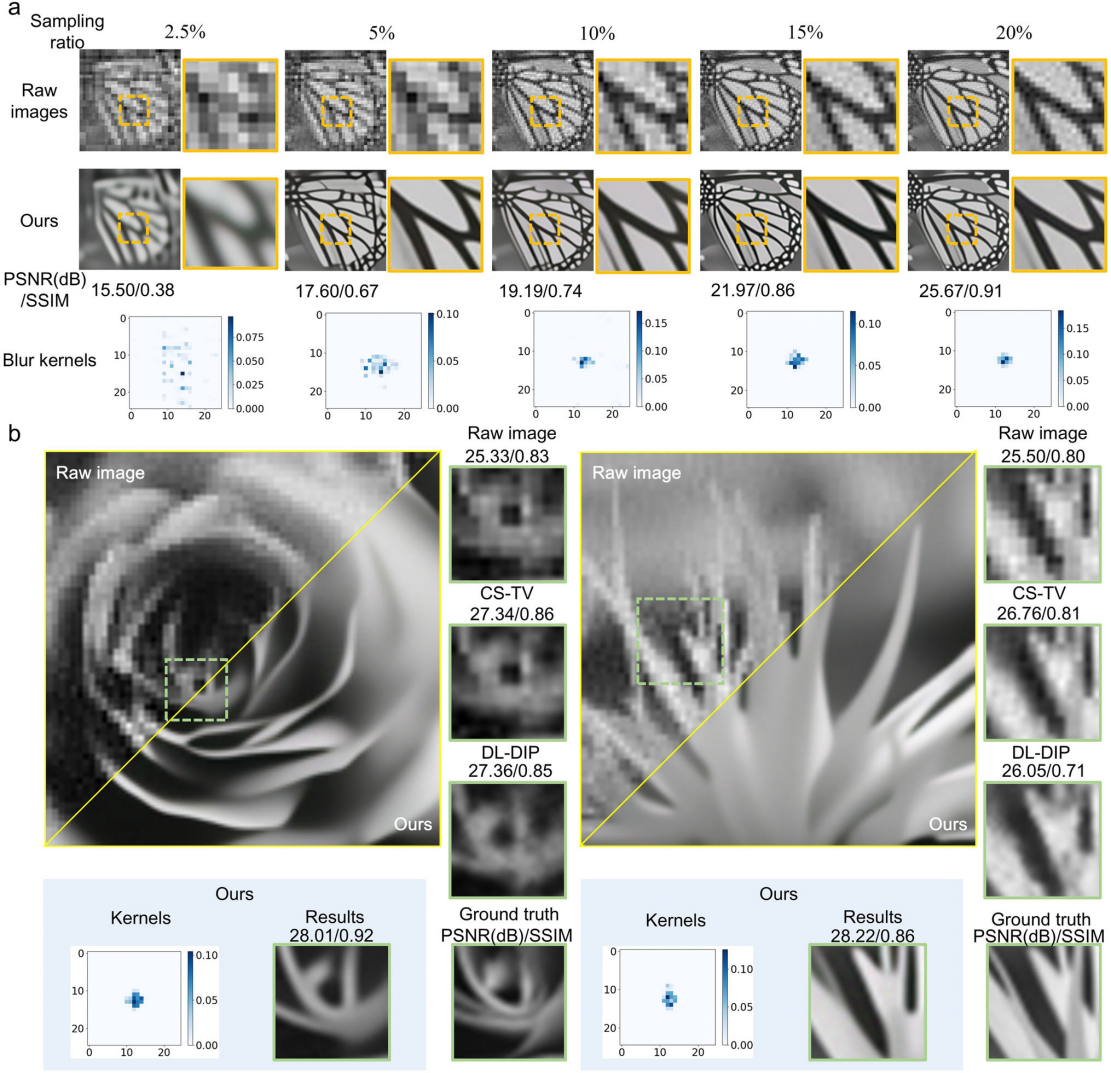
Simulation results for SPI using explicit compression degradation estimations. a) Blur kernels of compression degradation estimations and corresponding results at different sampling ratios. The kernels are calculated by the self‐supervised neural network using raw images and LSR images. b) Comparisons of simulation results from our compression degradation estimation method and typical model‐driven methods. PSNR and SSIM of entire images from different methods are listed above the enlarged views. The entire results are provided in Note S3 (Supporting Information).

We conducted simulations to validate the effectiveness of our degradation estimation technique for compressive SPI. As depicted in **Figure** **2**a, blur kernels of degradation estimations and results were calculated by the self‐supervised approach using raw images and LSR images without fully sampled results and any prior knowledge. Owing to spatial information reduction varying with sampling ratios, the reduction factor *s* was properly selected for various images in our estimation method (see Note S2, Supporting Information). For the object image in Figure 2a, we adopted *s* = 2 for results at sampling ratios of 15% and 20% and *s* = 4 for results at sampling ratios from 2.5% to 10%. Blur kernels in compression degradation estimations vary with sampling ratio δ and reduction factor *s* (see Note S2, Supporting Information). Based on estimated kernels, corresponding results with clear boundaries are reconstructed by the deconvolution network. Moreover, the peak signal‐to‐noise ratio (PSNR) and structural similarity index (SSIM) of the results are provided.

We compared our self‐supervised degradation estimation method with typical model‐driven SPI methods, including the compressive sensing method using total variation regularization (CS‐TV)^[^ ^15^ ^]^ and the deep learning method based on deep image prior (DL‐DIP).^[^ ^26^, ^27^ ^]^ The details of DL‐DIP are provided in Note S4 (Supporting Information). The above methods were performed on Hadamard SPI samples. Results and zoom‐in regions using measurements with a sampling ratio of 15% are presented in Figure 2b, where *s* = 2. Our results demonstrate that the inverse Hadamard transform approach fails to recover image details with clean edges. The methods using CS‐TV and DL‐DIP enhance the clarity of the boundary in SPI results. Still, the edges in images are blurred. In comparison, our degradation estimation method utilizes a self‐supervised approach to calculate blur kernels for compressive Hadamard SPI, which recovers images with better details and smoother surfaces. At the same sampling ratio, the estimated kernels for objectives with diverse structural information are various.

**Figure 3:**
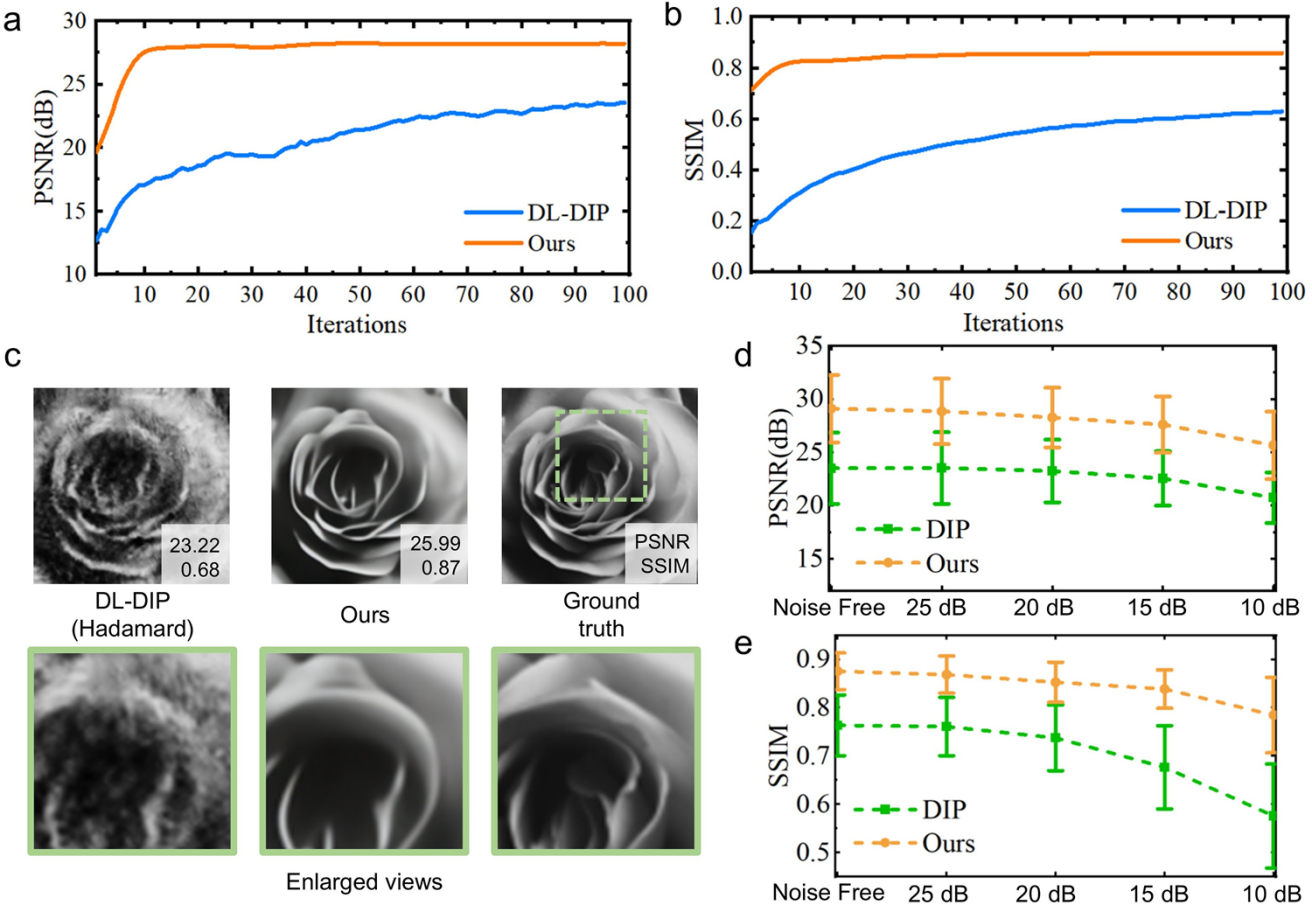
Numerical results of simulations. a, b) PSNR and SSIM of results varying with iterations. c) Reconstruction results and enlarged views from 1D measurements with noise. More results are provided in Note S5 (Supporting Information). d, e) Metrics of corrupted results under different noise levels.

As shown in **Figure** **3**a,b, the algorithm of our degradation estimation method would converge within 20 iterations, where the evaluation indices of results are stabilized. Results at various steps are provided in Video S1 (Supporting Information). To validate the robustness of our degradation estimation method for Hadamard SPI, we implemented simulations under noise. We added Gaussian white noise calculated by signal‐to‐noise ratio (SNR) to bucket measurements.^[^ ^35^ ^]^ Figure 3c displays the generated results from measurements disturbed by noise with an SNR of 10 dB. Compared to DL‐DIP, our method using pixel extraction shows better noise resistance performance. Moreover, we used 20 natural images to numerically test anti‐noise performance. The PSNR and SSIM of the results under various noise levels are plotted in Figure 3d,e. While image quality decreases as the noise level increases, our method still recovers results with better quality, especially SSIM. Image downsampling operations in our method may properly decrease noise interference for image restoration, resulting in better image quality. Overall, the above simulation results demonstrate that compression degradation estimations obtained by our method enhance the reconstruction quality and robustness of compressive SPI using the Hadamard basis.

### 2.3 Experiments

**Figure 4:**
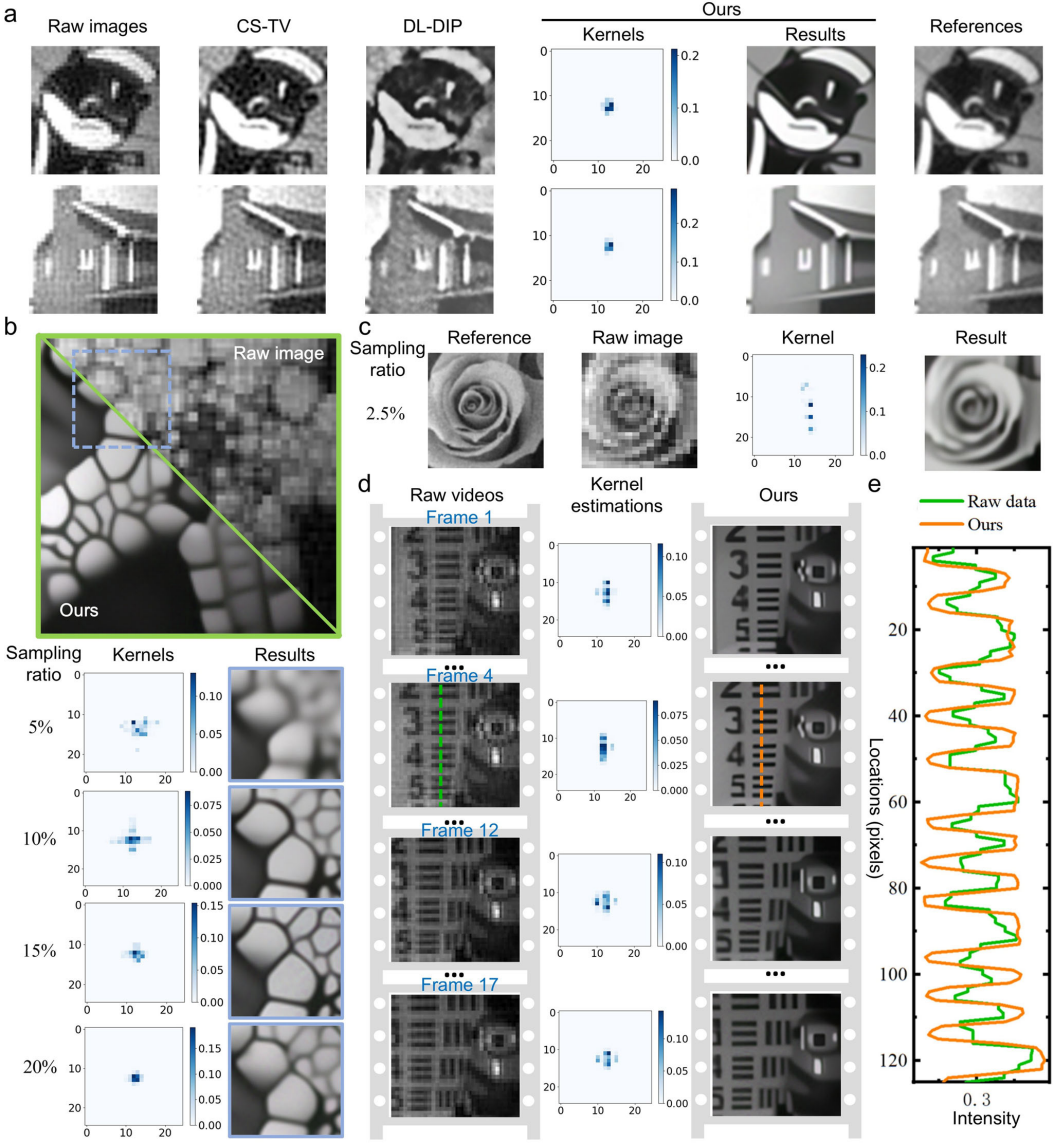
Experimental results of compression degradation estimations and reconstructions. a) Kernel estimations and images with a size of 64 × 64. b) 128 × 128 results at different sampling ratios. The entire results are provided in Note S6 (Supporting Information). c) Low‐sampling SPI results at a sampling ratio of 2.5%. d) Recovered frame images and kernel estimations for single‐pixel video imaging. e) Cross‐sectional profiles of dash lines.

We constructed an SPI device based on structured illumination for experiments (Experimental section and Note S7, Supporting Information). In the experiments, the proposed self‐supervised approach was used for compression degradation estimations. As shown in **Figure** **4**a, we employed our estimation method and typical model‐driven methods to reconstruct 64 × 64 normalized images of printed targets. We used spatially filtered results from fully sampled measurements as 2D references. Under a sampling ratio of 20% and a reduction factor of 2, we estimated blur kernels for compression degradation and generated SPI results with more image details and less noise. Figure 4b illustrates results with a size of 128 × 128 are recovered by our estimation method based on measurements with various sampling ratios from 5% to 20%. Moreover, the results at a low sampling ratio of 2.5% are shown in Figure 4c. We set the reduction factor *s* = 2 for results at sampling ratios from 10% to 20% and *s* = 4 for results at sampling ratios of 5% and 2.5%. The experiment results indicate that the proposed method using explicit compression degradation estimations enables more precise representation of the spatial image structures in low‐sampling SPI.

We experimentally conducted single‐pixel video imaging using our proposed estimation method. We used a DMD with a refresh rate of 10 kHz to project Hadamard patterns. In the first scene where a toy moved, we captured 128 × 128 single‐pixel image sequences at a sampling ratio of δ = 6.25%. We performed frame‐by‐frame video reconstruction by using the self‐supervised learning mechanism and the deconvolution network. Figure 4d displays 4 frames from the 128 × 128 single‐pixel video post‐processed by our method with *s* = 2. Furthermore, the cross‐sectional profiles of images are illustrated in Figure 4e. Compared to raw videos generated by inverse Hadamard transform, our results with better details and sharper edges are recovered by the deconvolution algorithm using corresponding kernel estimations. The 128 × 128 video with full frames is available in Video S2 (Supporting Information). In the dynamic scene with moving objects, the estimated kernels for video sequences are temporally variant. The kernels are associated with spatial textures at each position and motion information in each image frame.

## 3 Discussion

In summary, we have presented a degradation estimation technique to explicitly describe compressive sampling of low‐sampling SPI using the Hadamard basis. Using 2D results at different sampling ratios, we report a self‐supervised learning method to estimate blur kernels of the compression degradation models without labeled data and implicit priors. Our method incorporates a deconvolution network that recovers enhanced SPI results using blur kernels without training via Hadamard SPI datasets. Single‐pixel simulations and experiments verify that our approach is able to estimate blur kernels of explicit compression degradation models and outperforms typical model‐driven SPI methods in terms of reconstruction quality.

We discuss the novelty of the proposed SPI reconstruction method over typical model‐driven methods. First, we introduced the concept of explicit compression degradation estimations into SPI. SPI using insufficient measurements is a typical ill‐posed linear inverse problem. In existing model‐driven methods, several prior assumptions are employed to solve the inverse problem. Explicit compression degradation models of SPI were seldom investigated. Based on Hadamard basis patterns, the compression process leads to the loss of high‐frequency pixel information and mosaic artifacts, which are similar to super‐resolution issues rather than denoising issues. The degraded images are regarded as the ground truth after convolution with blur kernels. Second, we implemented a self‐supervised learning scheme for Hadamard SPI, which optimized neural networks using degraded images at different sampling ratios. In recent years, machine learning techniques have been employed to recover 2D results with better quality under a few measurements. Compared to existing model‐driven methods, our method generated kernel estimations of compression degradation models and results without any prior assumptions. Comparisons of estimated kernels from approaches using LSR images and fully sampled results are provided in Note S8 (Supporting Information). Additionally, our method is used to alleviate ringing‐like noise in Fourier SPI (see Note S9, Supporting Information).

Several improvements of the degradation estimation technique for compressive SPI are envisioned. First, we adopted an off‐the‐shelf model as the deconvolution network for image reconstruction. It is difficult to restore severely corrupted image structures in raw data by our method. Combining more advanced off‐the‐shelf models will further improve the quality of SPI results. For instance, generative models such as diffusion models^[^ ^23^, ^36^ ^]^ may generate more details to improve visual appearance. But it is difficult to avoid introducing the artificial details unrelated to the target. Second, we used a computer to perform the above algorithms. Using integrated platforms to implement algorithms may enhance the computation speed of image reconstruction and anti‐interference ability.^[^ ^37^, ^38^, ^39^ ^]^ Third, we presented the proposed method for 2D imaging applications. It can be extended to non‐imaging tasks,^[^ ^40^, ^41^, ^42^ ^]^ 3D imaging systems,^[^ ^13^, ^14^, ^43^, ^44^ ^]^ and non‐line‐of‐sight imaging approaches.^[^ ^45^, ^46^, ^47^ ^]^

## 4 Experimental Section

### Experimental Setup

The physics model of the experimental setup and its photograph are shown in Figure 1c and Figure S6 (Supporting Information), respectively. The experimental setup consisted of a light modulation part and a light detection part. In the modulation part, a light‐emitting diode operating at 400–760 nm (Daheng Optics, @ 20 W) was used as the light source of the light modulation part. A DMD (ViALUX V‐7001) with a projection module (Aunion Tech) modulated light from the light source. The light passing through a lens with a focal length of 300 mm illuminated objects. In the light detection part, the reflected light from objects was received by a bucket detector (Thorlabs PDA100A). Moreover, a data acquisition device (PicoScope‐6404E) collected the electrical signal from the bucket detector.

### Hadamard SPI Model

In Hadamard SPI systems,^[^ ^48^, ^49^ ^]^ Hadamard basis patterns Φ(*x*, *y*) are generated by:

(2) $$\Phi \left(x,y\right)=\frac{1}{2}\left[1+H^{-1}\left\{\varsigma (u,v)\right\}\right]$$

where ς(*u*, *v*) is a delta function and H^−1^{ · } is the inverse Hadamard transform. In this work, the Hadamard matrix based on sequency ordering^[^ ^50^ ^]^ with a zigzag scan was adopted.

Based on the Hadamard basis patterns Φ(*x*, *y*), the measurements *B_i_* for a target image *I*(*x*, *y*) are expressed as:

(3) $$B_{i}=\int \Phi (x,y)I\left(x,y\right)dxdy.$$

Limited by elements “0” and “1” in projection masks loaded on the DMD, differential Hadamard SPI was performed to acquire measurements. The differential Hadamard SPI uses measurements *B*_+_ and *B*_−_ based on the Hadamard basis patterns Φ_+_(*x*,*y*) and Φ_−_(*x*,*y*). The measurements are further written as:

(4) $$B=B_{+}-B_{-}.$$

The raw image $\hat{I}$ calculated by the inverse Hadamard transform is expressed as:

(5) $$\hat{I}=\Phi^{-1}B.$$

### The Reported Algorithms for Degradation Estimations and SPI Reconstructions

For the self‐supervised learning method in the kernel estimator *f*_θ1_, the blur kernel $\hat{k}$ in explicit degradation estimations can be obtained by solving:

(6) $$\arg\min_{\theta 1}{\left\|{\left({\hat{I}}_{↓s}⊗f_{\theta 1}\left(k_{0}\right)\right)}_{↓s}-{\hat{I}}_{l↓s^{2}}\right\|}^{2}$$

where ↓*s* ^2^ denotes the s^2^‐fold resolution reduction and *k*_0_ is the initial kernel estimation. The blur kernel $\hat{k}$ is written as:

(7) $$\hat{k}=f_{\theta 1}\left(k_{0};\hat{I};{\hat{I}}_{l}\right).$$

For the deconvolution network *f*_θ2_, the image restoration process is expressed as:

(8) $$\arg\min_{I}\frac{1}{2\sigma^{2}}{\left\|\hat{I}-\left(I⊗k\right){↓}_{s}\right\|}^{2}+\lambda \Phi \left(I\right)$$

where λ denotes a trade‐off parameter and σ is the standard deviation (or noise level).

In the training process, the deep unfolding network using a half‐quadratic splitting algorithm^[^ ^34^ ^]^ is adopted in the off‐the‐shelf model. An auxiliary variable *z* is introduced into the half‐quadratic splitting algorithm. The loss function can be written as:

(9) $$L_{DUN}\left(I,z\right)=\frac{1}{2\sigma^{2}}{\left\|\hat{I}-\left(z⊗k\right){↓}_{s}\right\|}^{2}+\lambda \Phi \left(I\right)+\frac{\mu }{2}{\left\|z-I\right\|}^{2}$$

where μ represents a penalty parameter.

The optimization problem can be solved by:

(10) $$\left\{\begin{array}{c} z_{i}=\arg\min_{z}{\left\|\hat{I}-\left(z_{i}⊗k\right){↓}_{s}\right\|}^{2}+\mu \sigma^{2}{\left\|z_{i}-I_{i-1}\right\|}^{2}\\ I_{i}=\arg\min_{I}\frac{\mu }{2}{\left\|z_{i}-I\right\|}^{2}+\lambda \Phi \left(I\right) \end{array}\right.$$

The algorithm of the deconvolution network *f*_θ2_ consists of a data module D and a denoiser P, which can be expressed as:

(11) zi=D(Ii−1,s,k,I^,μiσ2),Ii=P(zi,λλμiμi).

In the usage process, the inputs of the deconvolution network are the scale factor *s*, noise level σ, blur kernel $\hat{k}$, and raw image $\hat{I}$ via inverse Hadamard transform. Most notably, a released model with 8 iterations was employed as the off‐the‐shelf deconvolution network without training via Hadamard SPI datasets. The noise level σ ranges from 0.1 to 0.4, which depends on the sampling ratios of measurements.

The final Hadamard SPI reconstructions are written as:

(12) $${\hat{I}}_{k}=f_{\theta 2}\left(\hat{I},\hat{k}\right).$$

### Neural Networks and Hyperparameters

In this work, algorithms were run on a computer with an AMD Ryzen 7 5800H CPU and an Nvidia RTX 3060 GPU with 6 GB of memory. The network architecture for the kernel estimator was a fully connected network (see Note S10, Supporting Information). The kernel size of blur kernels was set to 25 × 25. In the deconvolution network, a network architecture with residual blocks and a U‐Net was used in the denoiser. With unaltered models, the parameters of the deconvolution network in the method remained consistent with those in the previous work. The adaptive moment estimation (Adam) optimizer with a learning rate 4 × 10^−4^ in the kernel estimator was adopted. A step decay with a decay rate of 0.8 and a decay step number of 10 was used for the optimizer. The kernel estimator for 100 iterations was performed.

## Conflict of Interest

The authors declare no conflict of interest.

## Supporting information



Supporting Information

Supporting Information

Supporting Information

## Data Availability

The data that support the findings of this study are available from the corresponding author upon reasonable request.
