# Supplementary material for: Explicit Compression Degradation Estimations for Low‐Sampling Single‐Pixel Imaging using Hadamard Basis
Source: Adv Sci (Weinh). 2025 Nov 30;13(9):e12655. doi: 10.1002/advs.202512655 (PMC12904066; doi:10.1002/advs.202512655)
Supplement: Supplementary file 1 — Supporting Information [file ADVS-13-e12655-s003.docx]

Supporting Information

Explicit compression degradation estimations for single-pixel imaging using Hadamard basis

*Haoyu Zhang,* *Jie Cao, * Chang Zhou, Haifeng Yao, and Qun Hao **

H. Zhang, J. Cao, C. Zhou, H. Yao, and Q. Hao

School of Optics and Photonics, Beijing Institute of Technology, Beijing, 100081, China
E-mail: ajieanyyn@163.com; qhao@bit.edu.cn

J. Cao, and H. Yao
Yangtze Delta Region Academy, Beijing Institute of Technology, Jiaxing, 314003, China

J. Cao
National Key Laboratory on Near-Surface Detection, Beijing, 100072, China

Q. Hao
Changchun University of Science and Technology, Changchun, 130022, China

**Contents**

**Note S1:** **The role of reduction factor** $s$

The role of reduction factor in the kernel estimator is described.

**Note S2: The value of reduction factor** $s$

The results using different reduction factors are shown.

**Note S3:** **Entire images of simulation results**

Entire images of simulation results are shown.

**Note S4: Details of DL-DIP**

The details of DL-DIP are described.

**Note S5:** **Simulation results under noise**

The simulation results under noise with various levels are shown.

**Note S6:** **Entire images of** **experimental results**

Entire images of experimental results are shown.

**Note S7: Experimental setup**

The photograph of experimental setup is shown.

**Note S8: Kernels from LSR images and fully sampled images**

Comparisons of estimated kernels from approaches using LSR images and fully sampled images are described.

**Note S9: Our method for Fourier SPI**

The results of Fourier SPI enhanced by our method are shown.

**Note S10: Structure of the fully-connected network in the kernel estimator**

The network structure of the kernel estimator is shown.

**Note S1:** **The role of reduction factor** $s$


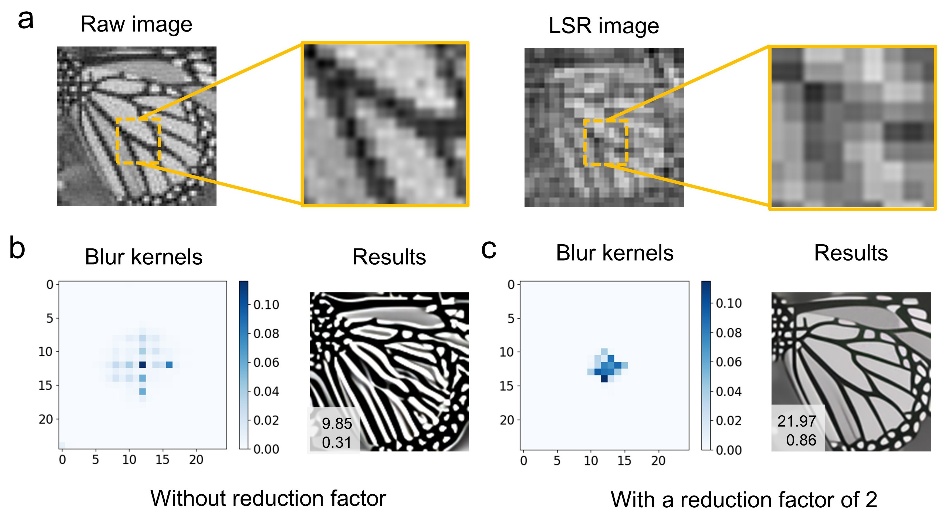
In single-pixel imaging (SPI) using Hadamard basis, mosaic artifacts exist in results from insufficient measurements. The kernel estimator of our method used a raw image $\hat{I}$ from $m$ measurements and a lower-sampling-ratio (LSR) image $\hat{I}_{l}$ from $m_{l}$ measurements to generate blur kernels. Following the relationship of sampling ratios in our proposed method, the sampling ratio of raw data was 15% and the sampling ratio of LSR data was set to 2.25%. As shown in Figure S1(a), slight mosaic artifacts and doubly degraded mosaic artifacts occur in raw images and LSR images. Figure S1(b) shows the inaccurate kernel estimation and the corresponding deconvolution result when using images without reduction factors. Figure S1(c) illustrates the kernel estimation and the result under a reduction factor $s$ of 2. Thus, the pixel extraction with a reduction factor $s$ avoids mosaic artifacts influencing blur kernel estimations.

**Figure S1.** **The role of reduction factor** $s$. (a) Entire views and zoomed views of the raw image and the LSR image. (b) Kernels and results from the approach using raw data without reduction factors. **c** Kernels and results from our method using a reduction factor of 2.


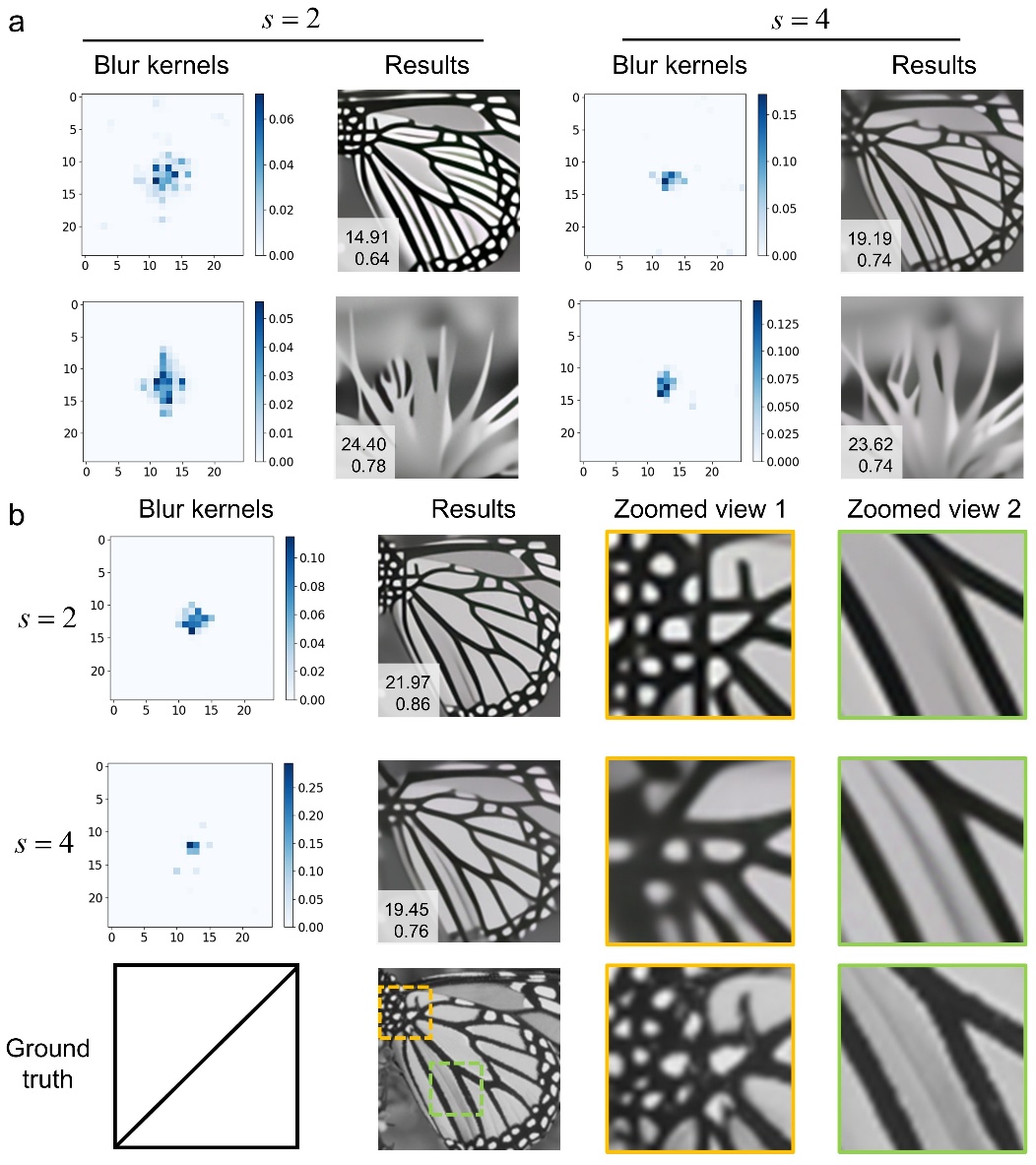
**Note S2: The** **value of reduction factor** $s$

**Figure S2.** **Results under different values of** **reduction factor** $s$. (a) Results of different objects under different reduction factors. Metrics (PSNR and SSIM) of entire images are provided. (b) Kernels and results varying with reduction factors.

Reduction factors affect the quality of results, which are properly selected in the kernel estimator. Different reduction factors are used for various image targets. Results of various images under different reduction factors are shown in Figure S2(a). We used measurements with a sampling ratio of 10% for reconstruction. Under the same sampling ratio, different reduction factors are suitable for various images. The quality of the result “Butterfly” under a reduction factor of 4 is better than that under a reduction factor of 2. But the consequent for the object “Grass” is not established. For the object “Grass”, the result with better details is recovered by our method using a reduction factor of 2. Accordingly, the quality of the result “Grass” under a reduction factor of 2 is better than that under a reduction factor of 4. Thus, we adopted $s=2$ for the object “Grass” and $s=4$ for the object “Butterfly” at a sampling ratio of 10%.

The evaluation metrics consist of PSNR [1] and SSIM [2]. Given a result $A$ and a reference image $B$, PSNR and SSIM are defined as

$$\text{SSIM}=\frac{(2\mu_{A}\mu_{B}+\zeta_{1})(2\sigma_{AB}+\zeta_{2})}{(\mu_{A}^{2}+\mu_{B}^{2}+\zeta_{1})(\sigma_{A}^{2}+\sigma_{B}^{2}+\zeta_{2})},$$

where $\mu_{A,B}$, $\sigma_{A,B}^{2}$ and $\sigma_{AB}$ are the means, variances, and covariance, respectively. The constants $\zeta_{1}$ and $\zeta_{2}$ are used to stabilize the computations.

Kernel estimations vary with the reduction factor $s$. We used measurements with a sampling ratio of 15% to reconstruct images of the object “Butterfly”. The kernels and results are shown in Figure S2(b). As the reduction factor $s$ increases, the area of kernels decreases. Images with more details are generated by our method using a reduction factor $s$ of 2. Thus, the reduction factor $s$ need to be set as small as possible while ensuring that results are not affected by mosaic artifacts. Excessive reduction factor $s$ will lead to more loss of details in raw images.

**Note S3:** **Entire images of simulation results**


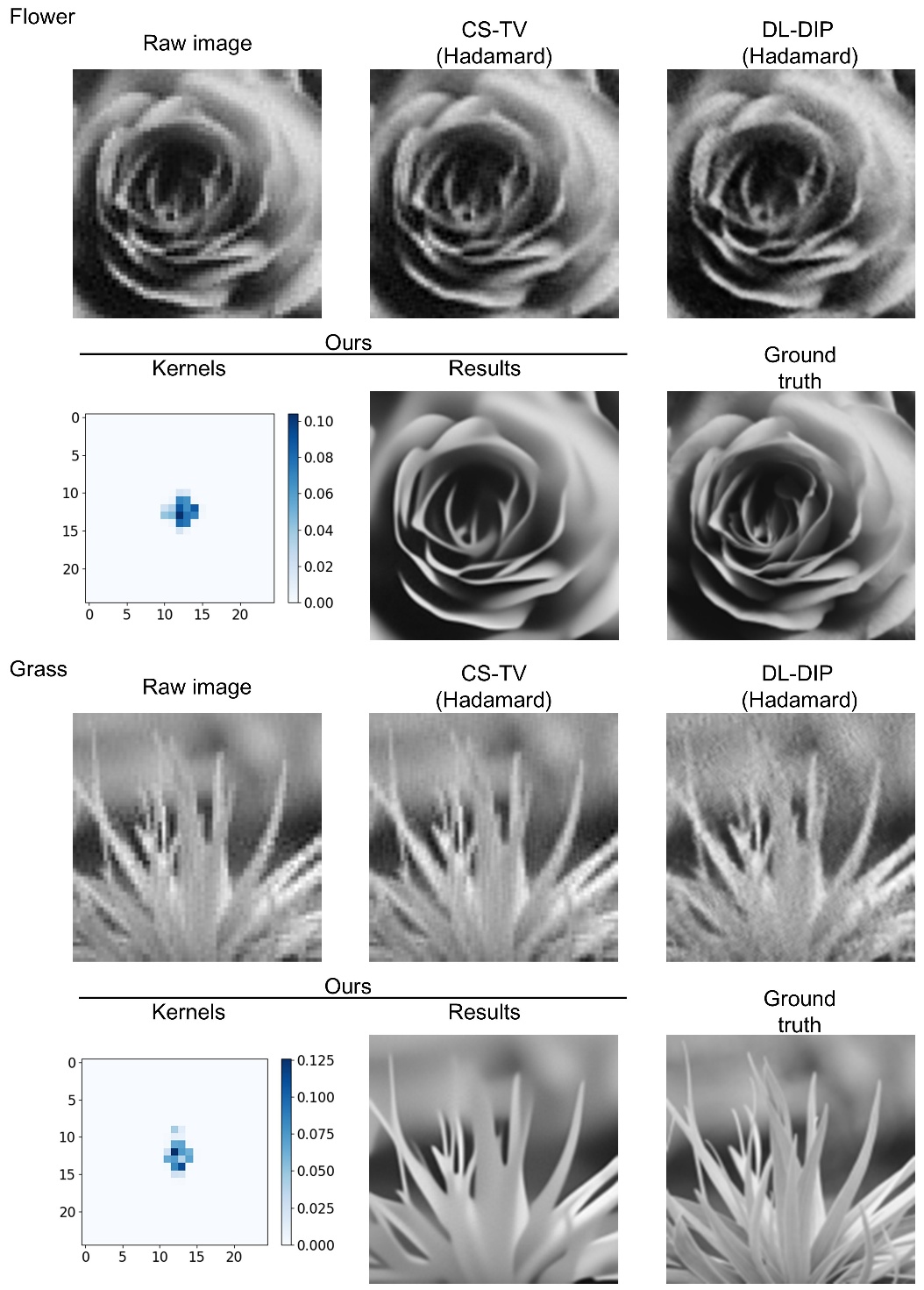
The entire images of simulation results are illustrated in Figure S3. Metrics (PSNR and SSIM) of entire images are provided in the manuscript.

**Figure S3.** **The entire simulation results**. Simulation results of objects “Flower” and “Grass” from different methods.

**Note S4: Details of** **DL-DIP**

We used the DL-DIP approach [3] using the constraint of measurements for comparison. We employed the encoder-decoder architecture [4] with a kernel size of $3\times3$ as the neural network in DL-DIP. The channel of the neural network was 32. The regularization parameter of total variation was set to $10^{-3}$. The learning rate for training with Adam optimizer was $10^{-4}$. We used a step decay with a decay rate of 0.5 and a decay step number of 200. The number of optimization iterations for training neural networks with DIP was set to 1000.

**Note S5:** **Simulation results under noise**


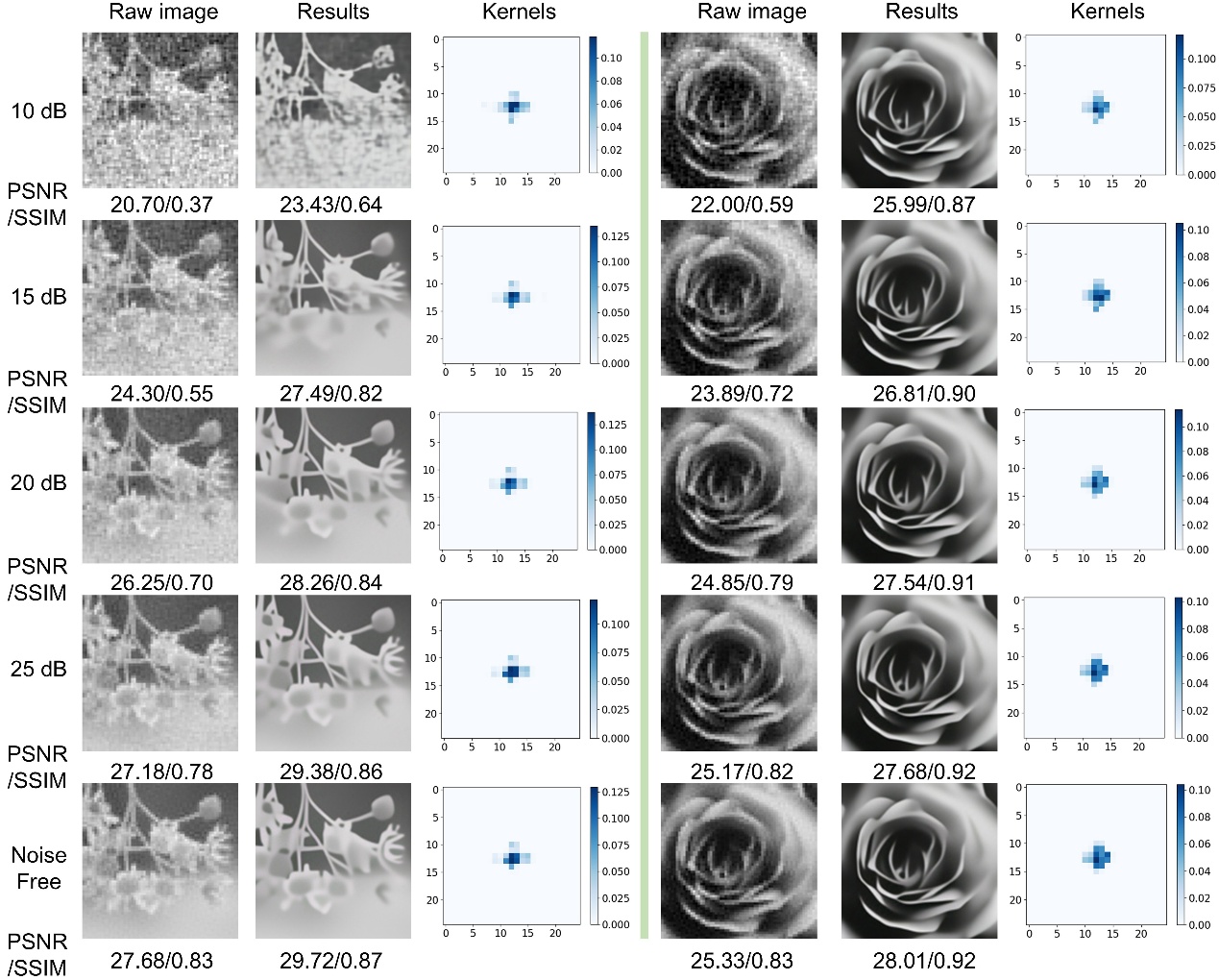
The results under noise with different levels are shown in Figure S4. The sampling ratio was set to 15%. We used our method with $s=2$ to estimate blur kernels and generate results. Kernel estimations under noise with different levels are similar and our results are slightly affected by noise compared to raw images.

**Figure S4.** **The simulation results under noise**. Kernel estimations and results of objects from our method.

**Note S6:** **Entire images of experimental results**


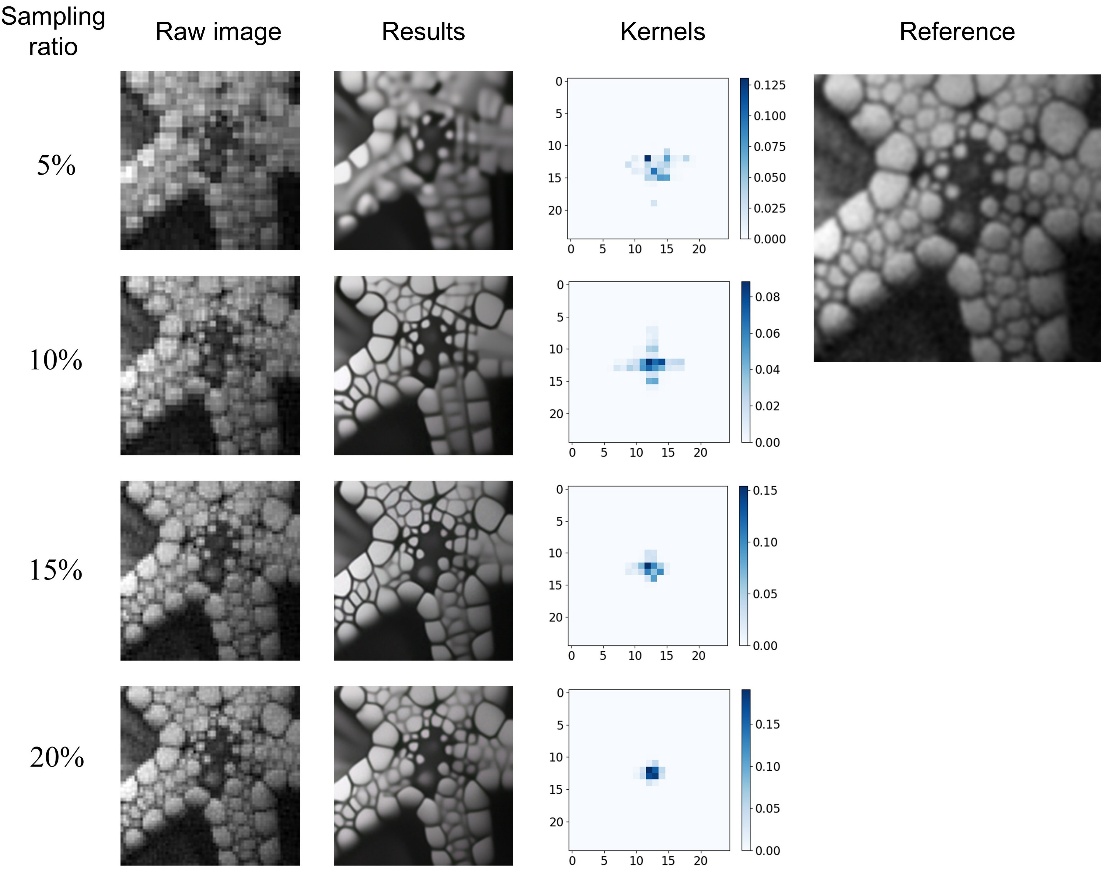
Figure S5 shows the entire images of experimental results from measurements with different sampling ratios. The image size was $128\times128$. Images with more details are recovered by the deconvolution network using kernel estimations.

**Figure S5.** **Entire images of** **experimental results**. Kernel estimations and results of experimental data with different sampling ratios.

**Note S7:** **Experimental setup**


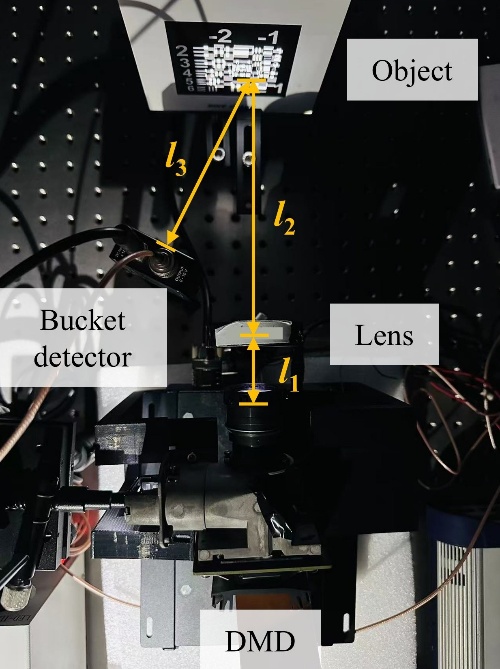
In the experiments, we built an experimental setup illustrated in Figure S6. We used a structured illumination scheme to conduct SPI experiments. A DMD with a projection module modulated the incident light from the light source. The object was illuminated by light passing through a lens. The distance $l_{1}$ between the projection module and the lens was 40 mm. The distance $l_{2}$ between the lens and objects was 165 mm. A bucket detector received the reflected light from objects. The distance $l_{3}$ between the bucket detector and objects was 120 mm. A data acquisition device collected the signal from the bucket detector.

**Figure S6.** **Experimental setup**. The SPI system mainly consists of a light source, a DMD, a bucket detector, a lens and a data acquisition device.

**Note S8: Kernels from LSR images and fully sampled results**


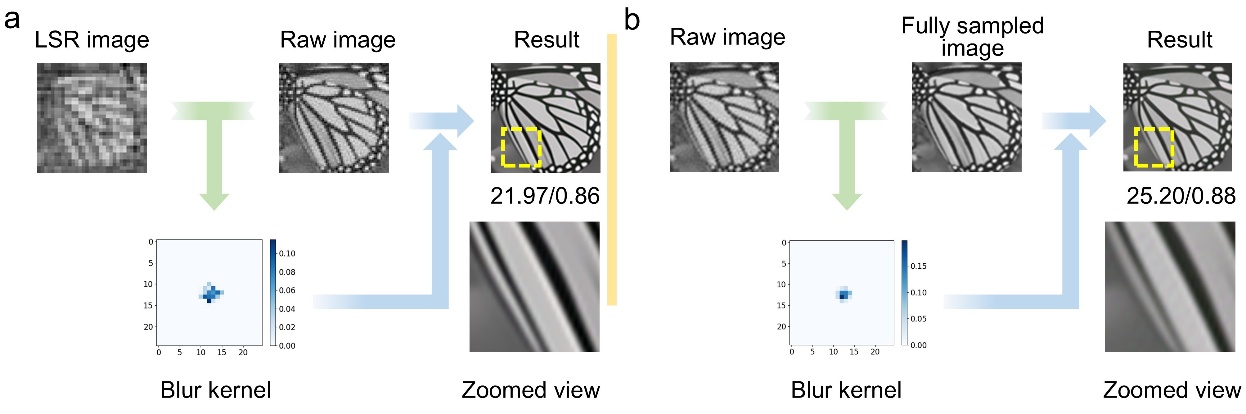
We estimated blur kernels by adopting approaches using LSR images and fully sampled results. Figure S7 shows the comparisons from different references. In our approach, LSR images and raw images are used to estimate blur kernels. Our results are illustrated in Figure S7(a). Compared to recovered results from the approach using raw images and fully sampled results shown in Figure S7(b), our results are recovered by using the kernel with a larger kernel area. The difference is reflected by image metrics. The PSNR of our results using LSR images is worse than that of results using fully sampled images, but results of two approaches have almost the same level of the SSIM for image structures. The worse PSNR is caused by the deconvolution algorithm using kernel estimations with inevitable deviation. The deviation of kernel estimations from the self-supervised approach affects the pixel values of image structures.

**Figure S7.** **Comparisons from** **approaches using LSR images and** **fully sampled images**. (a) Results from the reported method. (b) Results generated by using fully sampled images.

**Note S9: Our method for Fourier SPI**

We adopted our method to enhance compressive Fourier SPI [5]. In Fourier SPI, the Fourier spectrum of the object is acquired by measurements. The raw images are reconstructed by applying an inverse Fourier transform. The two-dimensional Fourier transform $F\{\cdot\}$ of an image $I(x,y)$ can be defined as

$$I_{F}(u,v)=F\{I(x,y)\}=\sum\sum I(x,y)\mathrm{e}^{-j2\pi(ux/M+vy/N)}.$$

Fourier basis patterns $\Phi_{F}(x,y)$ are obtained by applying an inverse Fourier transform $F^{-1}\{\cdot\}$ to a delta function , which can be expressed by

We employed the 4-step Fourier SPI as an example. Based on four measurements $B_{0}$, $B_{\pi/2}$, $B_{\pi}$, and $B_{3\pi/2}$, Fourier coefficients are calculated by

$$F(u,v)=(B_{\pi}-B_{0})+j(B_{3\pi/2}-B_{\pi/2}).$$

While using insufficient measurements, ringing-like noise [6] appears in the Fourier SPI results. We employed our method to alleviate ringing-like noise in compressive Fourier SPI. Compared to Hadamard SPI results with mosaic artifacts, Fourier SPI results are slightly disturbed by ringing-like noise. The compression degradation process in Hadamard SPI can be treated as super-resolution issues. However, the degradation phenomenon of compressive Fourier SPI is different from super-resolution issues. In the self-supervised learning scheme, the relationship of sampling ratios for Fourier SPI is different from that for Hadamard SPI. We use $m_{l}=m\cdot\tau$ for Fourier SPI, where $m_{l}$ and $m$ are the sampling numbers of raw images and LSR images. $\tau$ represents a scale factor. The results under different factors $\tau$ are shown in Figure S8. As $\tau$ increases, the image quality of results becomes better.


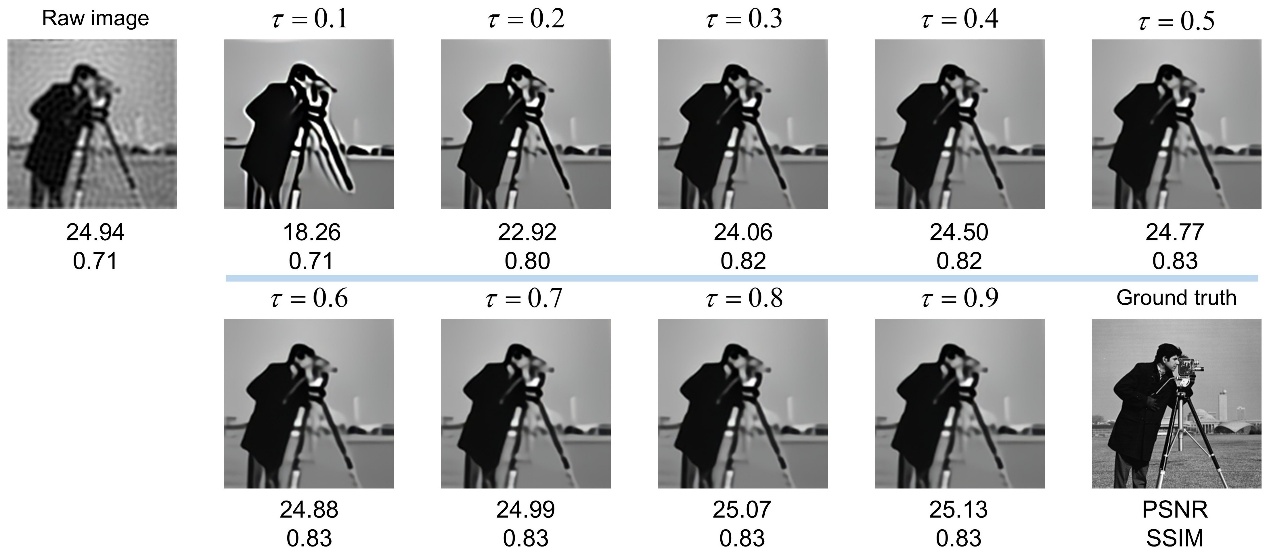


**Figure S8.** **Fourier SPI results enhanced by our approach**. 2D images under different scale factors are generated by self-supervised learning method using raw images and LSR images.

**Note S10: Structure of the fully connected network in the kernel estimator**

We employed a fully connected network for the kernel estimator in our method. The detailed network structure is illustrated in Figure S9. The input with a channel number of 200 was randomly generated. The channel numbers of the fully connected layer 1 and 2 were 1000 and 625. The fully connected layer 1 and 2 were separately followed by activated functions ReLU6 and Softmax. The channel of output was 625. The output was resized to $1\times1\times25\times25$.


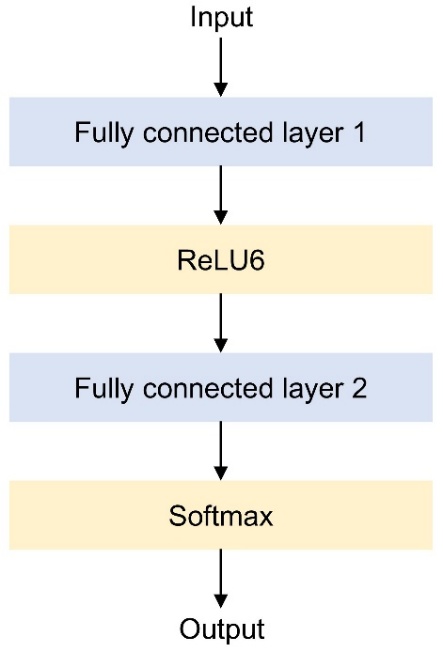


**Figure S9.** **Network structure of the fully connected network**. Only fully connected layers and activated functions are used in the network.

References

1. G. M. Gibson, S. D. Johnson, and M. J. Padgett, "Single-pixel imaging 12 years on: a review," Opt. Express **28**, 28190-28208 (2020).

2. W. Zhou, A. C. Bovik, H. R. Sheikh, and E. P. Simoncelli, "Image quality assessment: from error visibility to structural similarity," IEEE Trans. Image Process **13**, 600-612 (2004).

3. F. Wang, C. Wang, M. Chen, W. Gong, Y. Zhang, S. Han, and G. Situ, "Far-field super-resolution ghost imaging with a deep neural network constraint," Light Sci. Appl. **11**, 1 (2022).

4. O. Ronneberger, P. Fischer, and T. Brox, "U-Net: Convolutional Networks for Biomedical Image Segmentation," in *International Conference on Medical image computing and computer-assisted intervention*, 2015), pp. 234-241.

5. Z. Zhang, X. Ma, and J. Zhong, "Single-pixel imaging by means of Fourier spectrum acquisition," Nat. Commun. **6**, 6225 (2015).

6. Z. Zhang, X. Wang, G. Zheng, and J. Zhong, "Hadamard single-pixel imaging versus Fourier single-pixel imaging," Opt. Express **25**, 19619-19639 (2017).
